# Tocilizumab-Induced Dermatosis in a Patient With Rheumatoid Arthritis

**DOI:** 10.7759/cureus.32967

**Published:** 2022-12-26

**Authors:** Doaa Babkoor, Abeer Alshuqayfi, Nada Alshegaifi, Hanan Bamosa, Maryam Alsaid, Athba Alkinani, Shahad Algozi, Reema AlZaidi, Lina Alahmadi, Waleed A Hafiz

**Affiliations:** 1 Department of Dermatology, Alnoor Specialist Hospital, Makkah, SAU; 2 College of Medicine, Umm Al-Qura University, Alqunfudah, SAU; 3 College of Medicine, Taif University, Taif, SAU; 4 Department of Medicine, Alnoor Specialist Hospital, Makkah, SAU; 5 College of Medicine, Umm Al-Qura University, Makkah, SAU

**Keywords:** drug-induced reactions, dermatologic manifestations, dermatosis, tocilizumab, rheumatoid arthriitis

## Abstract

Rheumatoid arthritis is a chronic systemic autoimmune disease that results in symmetrical inflammatory polyarthritis with extra-articular involvement, including skin manifestations. It targets the lining of the synovial membranes and is treated with disease-modifying antirheumatic drugs. If left untreated, it leads to increased morbidity, mortality, and socioeconomic burdens. Tocilizumab is a humanized monoclonal antibody that binds to interleukin-6 receptors and is used to treat rheumatoid arthritis in patients with inadequate response to conventional synthetic therapy. This medication can cause adverse dermatologic events, such as urticaria, pruritus, and mild maculopapular rash. In this case, we report a 39-year-old woman with rheumatoid arthritis who developed tocilizumab-induced dermatosis.

## Introduction

Rheumatoid arthritis (RA) is a chronic systemic disease that causes inflammatory arthritis and leads to progressive, symmetric, and erosive destruction of cartilage and bones. It is an autoimmune disease characterized by the presence of autoantibody production, such as rheumatoid factor (RF) and antibodies against citrullinated peptides (anti-CCP). RA affects approximately 1% of the population. Other than inflammatory arthritis, patients may suffer from several extra-articular manifestations such as lung disease, cardiovascular disease, skin involvement, subcutaneous nodule formation, vasculitis, and inflammatory eye disease [1]., 

Skin is frequently involved in patients with RA, especially in the most severe and advanced forms of the disease. Skin manifestations in RA include rheumatoid nodules, rheumatoid vasculitis, pyoderma gangrenosum, rheumatoid neutrophilic dermatosis, and interstitial granulomatous dermatitis. Moreover, cutaneous reactions related to treatments of RA, in particular to biological agents, are all reported in the literature. Early recognition of these manifestations through clinical and histological assessment can expedite their diagnosis and management [2].

Currently, there are numerous medications for the treatment of RA. These medications are called disease-modifying antirheumatic drugs (DMARDs). They have both anti-inflammatory and immunomodulatory effects, thus slowing the disease process and preventing joint deformity. Among these medications is a medication called tocilizumab [3].

Tocilizumab is a monoclonal humanized anti-Interleukin 6 (IL-6) receptor antibody, which has been approved for the treatment of RA and has been shown to be highly and rapidly efficacious in improving the clinical, biochemical, and radiographic parameters of the disease. Tocilizumab reduces disease activity in RA by binding selectively to the increased soluble and membrane-bound IL-6 receptors (IL-6Rs) in the serum and synovial fluid [4].

Tocilizumab is generally safe with tolerable adverse reactions such as mild upper respiratory tract infections, high blood pressure, headache, hypercholesterolemia, and elevation of liver enzymes. Skin manifestations such as mild maculopapular rash, urticaria, and pruritus are infrequent but noticeable secondary to tocilizumab therapy [5].

Herein, we report a case of tocilizumab-induced dermatosis in a patient with RA.

## Case presentation

A 39-year-old woman, known to have type 1 diabetes mellitus for more than 25 years, presented to the rheumatology clinic with a four-month history of progressive inflammatory pain of multiple metacarpophalangeal (MCP) and proximal interphalangeal (PIP) joints bilaterally as well as wrists and ankles. She had no other symptoms of systemic rheumatic diseases. Physical examination revealed multiple tender and swollen MCP and PIP joints and left wrist, with no evidence of deformity. Cardiac, abdominal, neurologic, and dermatologic examination was unremarkable. Laboratory investigations are shown in Table 1. 

**Table 1:** Baseline laboratory investigations for the patient at the time of rheumatoid arthritis diagnosis

Test	Result	Reference range
Hemoglobin (Hb)	10.2 g/dL	12–15 g/dL
Platelets	329 × 10^9/L	150–400 × 10^9/L
White blood cells (WBC)	5.95 × 10^9/L	4–11 × 10^9/L
Erythrocyte sedimentation rate (ESR)	30 mm/h	3–10 mm/h
C-reactive protein (CRP)	10 mg/dL	0–0.3 mg/ dL
RF	97 IU/mL	0–15.9 IU/mL
Anti-cyclic citrullinated peptide (CCP)	Positive	Negative
Antinuclear antibodies (ANA)	Negative	Negative

Based on the laboratory investigations, she was diagnosed with seropositive RA with high disease activity and a Disease Activity Score (DAS)-28 C-reactive protein (CRP) score of 5.14. She was started on a short course of oral prednisolone at a dose of 15 mg daily with a quick taper, methotrexate 10 mg PO, and folic acid 5 mg PO once weekly. Her methotrexate dose was then increased gradually to 15 mg. Regular clinic follow-ups with her indicated complete resolution of her inflammatory arthritis with a DAS-28 CRP of 2.2 at nine months. However, she reported ongoing nausea and epigastric pain, which she attributed to methotrexate. These side effects interrupted her medication compliance several times. Modification in the treatment plan was discussed with her, methotrexate was discontinued, and tocilizumab was started at a dose of 4 mg/kg IV every four weeks. 

**Figure 1:**
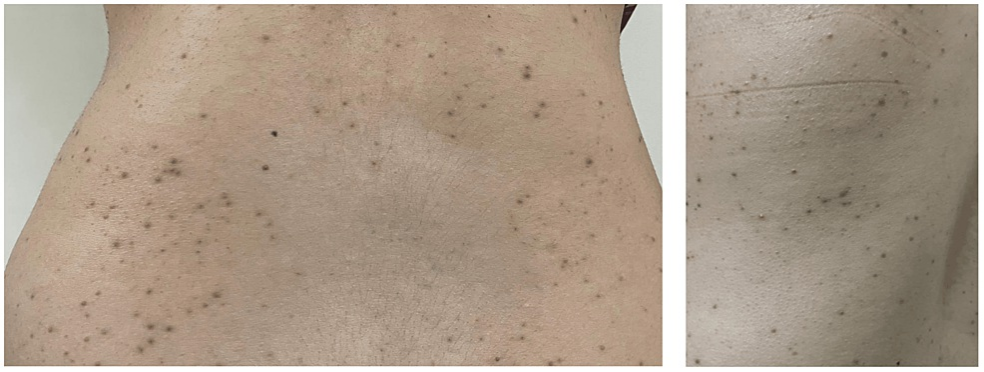
Papulosquamous cutaneous reaction on the trunk and arm with a few scratch marks and mild erythema

**Figure 2:**
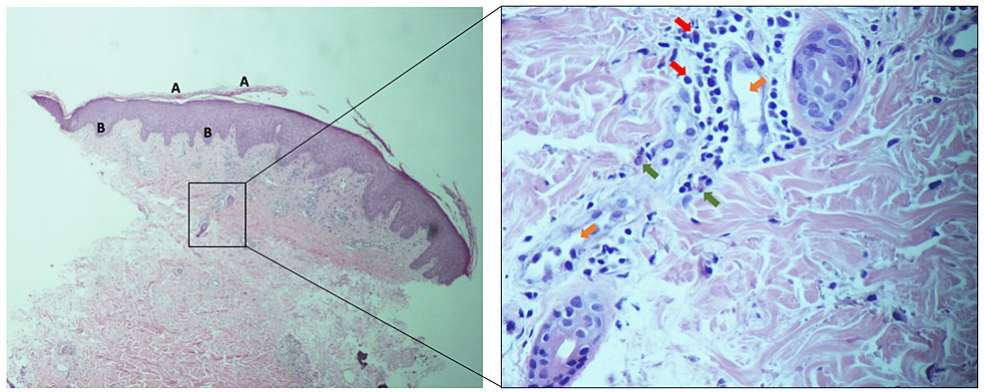
Skin biopsy showing hyperkeratosis (A) and acanthosis (B) with dilatation of blood vessels (orange arrows) and presence of numerous lymphocytes (red arrows) and eosinophils (green arrows)

After the second dose of tocilizumab, she presented to the clinic with new onset maculopapular rash over her back, abdomen, and arms with associated itching and redness but no blistering or discharge (Figure 1). She denied contact with any chemical substance or animal products preceding viral infection or administration of a new medication other than tocilizumab. She is not known to have an allergy to food, drugs, or environmental triggers. Tocilizumab was held, and a referral to dermatology for further assessment was initiated. Skin biopsy revealed orthokeratosis, hyperkeratosis, semiregular acanthosis with a prominent granular layer, dilated capillaries in the upper dermis with perivascular lymphocytic infiltrate, and a few eosinophils (Figure 2). For that, the clinical and histological findings were in keeping with drug-induced dermatosis secondary to tocilizumab. 

She returned to the rheumatology clinic after one month. Her skin rash resolved spontaneously; however, she was showing flare in her RA. Again, a treatment plan was discussed, and she decided to start tofacitinib at a dose of 5 mg PO twice daily, which brought her RA into remission in one month. 

## Discussion

Several dermatological adverse events are seen in patients taking tocilizumab [4]. These include injection site reactions, mild maculopapular rash, urticaria, pruritus, exanthematous pustulosis, and psoriasiform eruptions [5]. The majority of these manifestations are self-limiting and resolve upon tocilizumab discontinuation, and a few require topical corticosteroid therapy. Our patient developed tocilizumab-induced dermatosis.

Few cases of tocilizumab-induced skin reactions have been reported in the literature. The first case was a 79-year-old woman with seropositive RA for 20 years. She was treated for her highly active disease with gold, sodium thiomalate, hydroxychloroquine, leflunomide, azathioprine, methotrexate, etanercept, and adalimumab, all of which failed to control her disease activity. She was then commenced on tocilizumab plus oral prednisone. This treatment regimen resulted in a significant response, and her disease went into remission. After the sixth infusion of tocilizumab, she developed painful pustular psoriasiform eruptions on the back of both legs, the intergluteal cleft, behind the knees, and on the soles of her feet. Tocilizumab was discontinued, and these eruptions were treated with topical corticosteroids. Her skin rash improved after several weeks, but her arthritis flared eventually, after which she was started on certolizumab, with good clinical response and no recurrence of her rash [6]. The second case was a 55-year-old woman with adult-onset Still's disease. Her diagnosis was based on a history of spiking fevers, sore throat, arthralgia, evanescent maculopapular rash, leukocytosis, and elevated CRP and ferritin. Initial treatment with oral prednisolone and methotrexate failed to control her symptoms. For that, she was switched to tocilizumab monotherapy. Ten days later, she presented with a diffuse pruritic maculopapular rash. Laboratory evaluation revealed hyper-eosinophilia and elevations in the liver enzymes six times the upper limit of normal. Polymerase chain reaction test for human herpesvirus 6 was positive. Skin biopsy results showed lymphocytic and eosinophilic infiltration in the perivascular area. Tocilizumab was discontinued, and she was treated with topical corticosteroids. Skin rash resolved after five weeks of stopping tocilizumab [7]. Tocilizumab has also been linked to the development of possible Stevens-Johnson syndrome in a patient with giant cell arteritis [8], psoriasiform rash in a few patients [9], and drug-related generalized exanthematous pustulosis in a patient with RA [10].

To the best of our knowledge and according to the literature review, our case and the other above-mentioned cases demonstrate the development of dermatosis secondary to tocilizumab therapy.

## Conclusions

Tocilizumab-induced dermatosis is a rare but increasing dermatologic adverse event. The etiopathology of dermatosis during the IL-6 blockade is unclear but interesting and requires further exploration. This reversible adverse reaction needs to be considered when treating patients with tocilizumab.
